# Acute Effects of Handheld Loading on Standing Broad Jump in Youth Athletes

**DOI:** 10.3390/ijerph18095046

**Published:** 2021-05-10

**Authors:** Wei-Hsun Tai, Ray-Hsien Tang, Chen-Fu Huang, Shin-Liang Lo, Yu-Chi Sung, Hsien-Te Peng

**Affiliations:** 1 School of Physical Education, Quanzhou Normal University, Quanzhou 362000, China; dlove520@hotmail.com; 2 Graduate Institute of Sport Coaching Science, Chinese Culture University, Taipei 11114, Taiwan; 3 Department of Physical Education, National Taiwan Normal University, Taipei 106209, Taiwan; 295@htjh.tp.edu.tw (R.-H.T.); t08001@ntnu.edu.tw (C.-F.H.); 4 Department of Physical Education, Chinese Culture University, Taipei 11114, Taiwan; lxl6@ulive.pccu.edu.tw; 5 Department of Combat Sports and Chinese Martial Arts, Chinese Culture University, Taipei 11114, Taiwan; syq2@ulive.pccu.edu.tw

**Keywords:** kinematics, kinetics, biomechanics, coordination

## Abstract

The study aimed to investigate the acute effects of handheld loading on standing broad jump (SBJ) performance and biomechanics. Fifteen youth male athletes (mean age: 14.7 ± 0.9 years; body mass: 59.3 ± 8.0 kg; height: 1.73 ± 0.07 m) volunteered to participate in the study. Participants were assigned to perform SBJ with and without 4 kg dumbbells in a random order. Kinematic and kinetic data were collected using 10 infrared high-speed motion-capture cameras at a 250 Hz sampling rate and two force platforms at a 1000 Hz sampling rate. A paired *t*-test was applied to all variables to determine the significance between loading and unloading SBJs. Horizontal distance (*p* < 0.001), take-off distance (*p* = 0.001), landing distance (*p* < 0.001), horizontal velocity of center of mass (CoM; *p* < 0.001), push time (*p* < 0.001), vertical impulse (*p* = 0.003), and peak horizontal and vertical ground reaction force (GRF; *p* < 0.001, *p* = 0.017) were significantly greater in loading SBJ than in unloading SBJ. The take-off vertical velocity of CoM (*p* = 0.001), take-off angle (*p* < 0.001), peak knee and hip velocity (*p* < 0.001, *p* = 0.007), peak ankle and hip moment (*p* = 0.006, *p* = 0.011), and peak hip power (*p* = 0.014) were significantly greater in unloading SBJ than in loading SBJ. Conclusions: Acute enhancement in SBJ performance was observed with handheld loading. The present findings contribute to the understanding of biomechanical differences in SBJ performance with handheld loading and are highly applicable to strength and conditioning training for athletes.

## 1. Introduction

Before the 18th ancient Olympic Games in 708 BC, athletes used handheld weights with a sling, designed for an easy grip in the standing broad jump (SBJ). These primitive stone or lead dumbbells, which were called halteres and weighed 2–9 kg [1,2,3] were intended to extend the jumping distance. Depictions of athletes performing various SBJ maneuvers using these weights have been found on numerous artifacts, including pictographs [3,4].

Handheld loads used in SBJ, whether ancient or modern, are designed to improve the jumping distance [5,6,7,8,9]. Campa et al. [10] reported that the capacity of the explosive power of lower extremity was in correlation with the repeated-sprint ability (RSA) relate to jumping ability. Moreover, loading plyometric training has proven to be one of the more effective ways of improving sprinting and jumping performance [11]. Empirical and simulation studies [3,5,6,7,8,9] have reported increases of between 5 and 39 cm with handheld loading [3,7,8,12,13], with improvements in take-off velocity, horizontal distance of center of mass (CoM) at landing [7,8,13], and ground reaction force (GRF) or impulse [3,8,9,13].

Evidence indicates that SBJ performance improves under handheld loading [8,12,13,14]. Participants in relevant studies have included female netball players, physically active male university students, and university students in general. Notably, the loading intensity varies across these studies, with male participants’ handheld loads being greater than female participants’, and adults’ handheld loads being greater than those of youths. This is attributable to the differences in sex, age, motor skill development, and individual physiological variations [14,15,16,17]). In addition, athletic experience affects jumping performance [17].

Studies have indicated that the differences in SBJ performance under loading, including the changes in the aforementioned variables, are affected by both gender and the specific sport that athletes practiced. The acute effects of handheld loading on SBJ and their underlying mechanisms remain unclear, and the most relevant research only included adult participants. Therefore, the purpose of this study was to investigate the acute effects of handheld loading on the performance and biomechanics during SBJ, in youth athletes.

## 2. Materials and Methods

### 2.1. Participants

A total of 15 male track and field youth athletes volunteered to participate in this study (mean age: 14.7 ± 0.9 years; body mass: 59.3 ± 8.0 kg; height: 1.73 ± 0.07 m). A priori sample size calculation was performed using G*Power (version 3.1.9.2, Franz Faul, Universität Kiel, Germany), with a power level of 75% and an α level of 0.05. The expected effect size was calculated using means (206 and 223) and standard deviation (20 and 23) of the horizontal distance with loading and unloading SBJ. It shows that the sample size of 15 participants would be enough for the analysis. All participants were free from lower extremity injury six months prior to testing. All participants and their guardians provided written informed consent and the research obeyed the declaration of Helsinki.

### 2.2. Procedures

The procedures for the optimal handheld load testing session were similar to McKenzie et al. [1]. Each participant completed three jumps at each of the following total loads of two dumbbells: 0 kg (unloading), 1 kg, 2 kg, 3 kg, 4 kg, and 5 kg, to determine each participant’s optimal handheld load based on curve fitting for jumping performances. 4 kg showed an optimal jumping performance (each hand holding 2 kg dumbbells). Thereafter, the study focused on the biomechanical difference between SBJs with and without 4 kg dumbbells (loading and unloading). The participants were familiarized with the required jump movement prior to the testing session and performed a series of dynamic stretches and warm-up activities for 15 min. All participants performed loading and unloading SBJs in a random order. Three trials of each SBJ were collected for each participant. There was a 5 min rest between trials.

### 2.3. Experimental Instruments and Equipment

Kinematic and kinetic data were collected using ten infrared motion-capture cameras (Vicon MX 13+, Oxford Metrics Ltd., Oxford, UK) at a 250 Hz sampling rate and two force platforms (60 × 90 cm Kistler, Instruments, Inc., Winterthur, Swiss) at an 1000 Hz sampling rate. The kinematic and kinetic data were recorded using a motion capture and analogue data acquisition system (Nexus 1.4, Oxford Metrics Ltd., Oxford, UK). A total of 15 body segments (head, trunk, pelvis, and bilateral thigh, as well as calf, foot, upper arm, forearm, and hand) were defined by 69 retroreflective markers (19 mm in diameter), according to the modified Helen Hayes configuration.

### 2.4. Data Reduction and Analysis

Three-dimensional trajectory data of the markers and GRF data were identified within the Vicon and Kistler (Bioware 3.2, Kistler, Instruments, Inc., Winterthur, Swiss) softwares, respectively. Both were exported to a C3D file format and further imported into the Visual 3D software (C-motion, Rockville, MD, USA) to analyze the kinematics and kinetics data. The kinematic and kinetic data were filtered by a low-pass Butterworth digital filter at a cut-off frequency of 6 Hz, which was defined by means of the residual analysis method proposed by Winter [18] and was used to filter random noise during the digitizing process. The joint moment and power were calculated from the kinematic and GRF data, using inverse dynamics [18].

**Figure 1:**
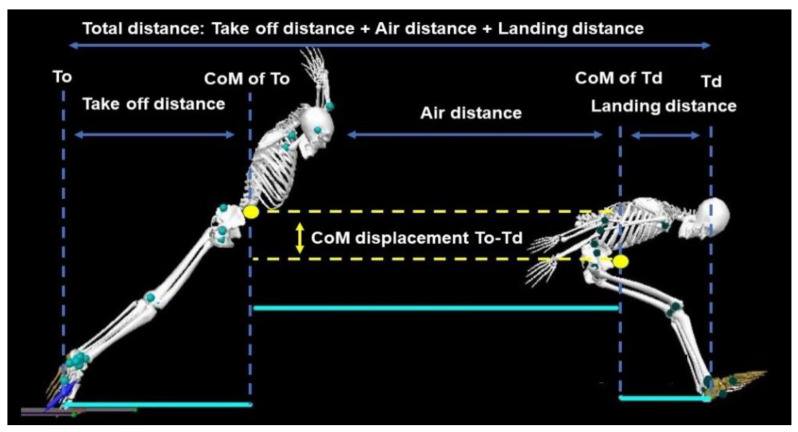
Definition of main distances in the standing broad jump. To = take-off; CoM = center of mass; Td = touch-down.

A take-off angle was calculated from the horizontal and vertical velocity of the jumper’s CoM at the instant of take-off according to the tangent of the trigonometric function. Push-off time was defined from when the start of downward and backward GRF exceeded 20 N to the instant of take-off. The impulse was calculated from the integration of the GRF–time curve. The measurement of the jumping performance of SBJ movement was separated into three parts: (1) take-off distance, the horizontal distance between the front edge of the toe, and the position of the jumper’s CoM at the instant of take-off; (2) air distance, the horizontal distance of CoM between the instant of take-off and touch-down of the ground upon landing; and (3) landing distance, the horizontal distance of CoM between the position of the jumper’s CoM and the rear edge of the heel at the instant of touch-down (Figure 1). The total distance was the summation of the take-off distance, air distance, and landing distance [19].

### 2.5. Statistical Analysis

Statistical analysis was performed using SPSS for Windows (IBM SPSS Statistics 20.0, Somers, New York, NY, USA). Descriptive data were expressed as the means (M) and standard deviations (SD) of the variables. The Normality was assessed using the Shapiro–Wilk test, and the Wilcoxon test was used when the data were not normally distributed, which was applied to all biomechanical variables in order to determine the significance between loading and unloading SBJs. The statistical level of significance was set at 0.05. The effect size (*r*^2^) for the differences was calculated to indicate the practical relevance of the significance. For *r*^2^, it was indicated that with a large effect is 0.5, a medium effect is 0.3, and a small effect is 0.1 [20].

## 3. Results

The total distance (*Z* = −3.352, *p* = 0.001, *r*^2^ = 0.749), take-off distance (*Z* = −2.884, *p* = 0.004, *r*^2^ = 0.539), landing distance (*Z* = −3.011, *p* = 0.003, *r*^2^ = 0.604), and horizontal velocity of CoM (*p* = 0.002) of loading SBJ were significantly greater than those of unloading. Moreover, the take-off vertical velocity of CoM (*Z* = −3.296, *p* = 0.001, *r*^2^ = 0.724); take-off angle (*Z* = −3.296, *p* = 0.001, *r*^2^ = 0.724); and peak angular velocity of ankle, knee, and hip (*Z* = −1.998, *p* = 0.047, *r*^2^ = 0.263; *Z* = −3.294, *p* = 0.001, *r*^2^ = 0.723; *Z* = −2.669, *p* = 0.008, *r*^2^ = 0.475) of loading SBJ were significantly smaller than those in unloading (Table 1).

The push-off time (*Z* = −3.411, *p* = 0.001, *r*^2^ = 0.776), vertical impulse (*Z* = −2.982, *p* = 0.003, *r*^2^ = 0.593), and peak horizontal and vertical GRF (*Z* = −3.296, *p* = 0.001, *r*^2^ = 0.724; *Z* = −2.341, *p* = 0.019, *r*^2^ = 0.365) of loading SBJ were significantly greater than those of unloading. Moreover, the peak ankle and hip moment (*Z* = −2.556, *p* = 0.011, *r*^2^ = 0.436; *Z* = −2.669, *p* = 0.008, *r*^2^ = 0.475) and peak hip power (*Z* = −2.499, *p* = 0.012, *r*^2^ = 0.416) of loading SBJ were significantly smaller than those of unloading (Table 2).

## 4. Discussion

In this study, the acute effects of handheld loading on SBJ biomechanics were investigated. The major finding were that, compared with unloading SBJ, the jumping distance, take-off distance, landing distance, vertical impulse, peak vertical GRF, and peak horizontal GRF increased by 8.25%, 12.5%, 50%, 14.6%, 7.3%, 19%, and 9.8%, respectively; however, the joint moment of the ankle, knee, and hip decreased by 7.5%, 9.3%, and 13.8%, respectively.

Overall, handheld loading can effectively increase SBJ distance in youth athletes. In the present study, the increased jump distance and take-off distance with handheld loading were in accordance with previous studies [7,8,15]. Moreover, an increased landing distance with handheld loading was observed. It was suggested that the handheld load altered the CoM positions before take-off and after landing, thereby enhancing the SBJ performance of youth athletes. One of the reasons could be the handheld loading increased horizontal take-off velocity, which leads to a reduced take-off angle, and resulting in the feet touch-down in front of the body [19]. Another reason could be that arm swing under appropriate loading conditions coordinates the body position before take-off and increases the take-off horizontal velocity [21,22,23,24], adjusting the lower-extremity mechanics [5,6,7,8,9].

In the present study, loading SBJ showed greater push-off time but smaller knee and hip joint angular velocity, compared with unloading SBJ. Previous studies have indicated that longer push-off phase was one of the key factors for good SBJ performances [16]. During the push-off phase of loading SBJ, the greater push-off time with slower knee and hip joint movement may help to increase the muscle activation in the lower extremities, allowing the coordination of an optimal take-off position [14] that could also produce greater GRF and impulse [15,21,25,26]. Compared with unloading SBJ, loading SBJ demonstrated greater vertical and horizontal GRF and vertical impulse, in the present study.

However, there was no significant increase in the horizontal impulse, which was inconsistent with the results of previous studies [15,27]. This may be due to individual differences in SBJ strategies of youth and adult athletes [14,18,28]. Youth athletes may not take enough SBJ practice before testing, or, may not be familiar with extra loads. With the decreased of arms swining motion during take-off, the body had to remedy it by excessive forward rotation during take-off [22], which means that the larger moments were destabilizing to offset the body’s forward rotation while the center of mass was redistributed during take-off by the extra load. In general, SBJ is more technical than the vertical jump during the push-off phase [29], and individuals may choose to apply more vertical or horizontal force to the push-off, depending on their lower extremity strength [8,14,27]. The extra loading on hands and the lack of lower limb strength can change the coordination of SBJ, and these perhaps are affected by age and technical ability [15,27].

Jumping is a movement that requires a sequence of coordination between body segments, and different jumping movements may be driven by different mechanisms. Previous studies have indicated that the arm motion in SBJ results in higher ankle and hip moment during countermovement jumps [26]; Moreover, drop jumps under higher loading result in a higher joint moment and power of the lower extremity [30]. By contrast, the results of lower extremity joint moment in the present study are inconsistent with those of previous studies [24,26]. The ankle and hip moment in loading SBJ were lower in the present study. The reductions in ankle and hip moment under loading may be partially ascribed to the fact that the load produced forward pulling after the arm swing. The larger moments of the lower extremity can lead to destabilization during SBJ, which disturbs the body’s rotation during SBJ [22]. In loading SBJ of youth athletes, the extra loading of hands pulls their body upward and forward with arm swing, which causes a decrease in the joint moment of the lower extremity, but an increase in the push-off duration and GRF, which helps with their energy transmission to the body during the push-off phase [31].

Previous studies have demonstrated that the pulling force of the loading involved in the arm swing affects joint mechanical output during jumps [22,26,31], and there was less power output in vertical jumps under greater loadings [32]. In the present study, the lower hip power generation in loading SBJ during the push-off phase was in accordance with these previous studies. The handheld load changed SBJ mechanism, necessitating a re-coordination of the muscle activation sequence, thus influencing the mechanical output [33]. Moreover, it may be due to the forward pulling of the load that the joint power generation decreases during loading SBJ.

There were some limitations in the present study. Only a 4 kg handheld load was used for the experiment, although some participants performed well with 3 kg and 5 kg loads. The strength of the lower extremity and jumping ability of the youth athletes were not measured prior to testing, which was a methodological limitation and an internal challenge for maturation. We conclude that handheld loading is helpful for SBJ training.; however, the optimal load should take into consideration individual differences before training, though 4 kg has been a proven application for the young athlete group in the present study.

## 5. Conclusions

In this study, the acute effects of handheld loading on the improvement of performance during SBJ in youth athletes were verified in biomechanics. When performing SBJ, athletes and their coaches could not pay attention to the joint biomechanics’ patterns. Meanwhile, this study revealed the effects of handheld loading on the biomechanics of the individual coordination of lower extremity during SBJ. Youth athletes’ hip moment and knee joint angular velocity were decreased by arm swing with extra loading from hands, which changed the take-off strategy to horizontal direction and enabled a longer push-off time to re-coordinate a better position and consequently to improve SBJ performance. The present findings contributed to the understanding of biomechanical knowledge in youth athletes’ SBJ performance with and without handheld loading, which can be used in their training program in order to improve their jumping technique, as it is highly applicable for their strength and conditioning training. Taken together, it is recommended that athletes and their coaches carefully select the handheld loads during SBJ, considering the individual differences of physical ability and coordination.

## Figures and Tables

**Table 1:** Kinematic variables in standing broad jumps.

Variables	Unloaded		Loaded		*Z*	*p* (95% CI)	*r* ^2^
	M	SD	M	SD			
Horizontal distance (cm) *	206	20	223	23	−3.352	0.001 (−22.29, −11.85)	0.749
Horizontal velocity of CoM (m/s) *	3.05	0.29	3.23	0.33	−3.108	0.002 (−0.25, −0.10)	0.644
Vertical velocity of CoM (m/s) *	1.84	0.21	1.56	0.21	−3.296	0.001 (0.13, 0.43)	0.724
Resultant velocity of CoM (m/s)	3.58	0.17	3.59	0.31	−0.597	0.551 (−0.13, 0.11)	0.024
Take-off angle (degree) *	29.64	4.22	24.95	3.60	−3.296	0.004 (2.80, 6.57)	0.724
Take-off distance (cm) *	56	7	63	6	−2.884	0.001 (−0.10, −0.03)	0.539
Air distance (cm)	125	12	124	14	−1.131	0.258 (−0.40, 0.07)	0.085
Landing distance (cm) *	24	5	36	9	−3.011	0.003 (−0.18, −0.06)	0.604
CoM vertical displacement To-Td (cm)	24	5	26	2	−1.762	0.078 (−0.47, 0.01)	0.207
Peak ankle angular velocity (°/s) *	588.7	86.9	561.3	73.2	−1.988	0.047 (−3.20, 58.05)	0.263
Peak knee angular velocity (°/s) *	684.5	66.4	627.9	59.9	−3.294	0.001 (30.31, 82.83)	0.723
Peak hip angular velocity (°/s) *	374.6	44.7	332.7	56.5	−2.669	0.008 (12.89, 70.75)	0.475

* Significant difference between loaded and unloaded SBJs (*p* < 0.05); CoM = center of mass, To = take-off, Td = touchdown.

**Table 2:** Kinetic variables in standing broad jumps.

Variables	Unloaded		Loaded		*Z*	*p* (95% CI)	*r* ^2^
	M	SD	M	SD			
Push-off time (ms) *	41	3	47	5	−3.411	0.001 (−7.76, −4.37)	0.776
Horizontal impulse (N·s)	125.7	19.9	134.2	23.5	−1.817	0.069 (−17.37, 0.36)	0.220
Vertical impulse (N·s) *	241.0	37.6	260.9	52.3	−2.982	0.003 (−32.84, −7.10)	0.593
Peak horizontal GRF (N) *	597.7	76.2	712.3	106.0	−3.296	0.001 (−148.66, −80.60)	0.724
Peak vertical GRF (N) *	1200.4	220.2	1307.1	213.5	−2.341	0.019 (−192.69, −20.49)	0.365
Peak moment (Nm)							
Ankle *	136.7	26.3	127.5	26.9	−2.556	0.011 (3.06, 15.31)	0.436
Knee	208.5	52.4	203.3	68.9	−1.477	0.140 (−13.23, 23.77)	0.145
Hip *	138.1	19.1	125.7	23.4	−2.669	0.008 (3.82, 21.03)	0.475
Peak power generation (Watt)							
Ankle	897.2	179.9	845.6	206.5	−1.590	0.112 (−7.66, 110.95)	0.169
Knee	1613.3	341.7	1667.1	485.9	−0.625	0.532 (−192.14, 84.72)	0.026
Hip *	1046.1	148.4	927.2	214.1	−2.499	0.012 (30.61, 207.19)	0.416

* Significant difference between loaded and unloaded SBJs (*p* < 0.05); GRF = ground reaction force.
